# The Impact of Thermal Water in Asthma and COPD: A Systematic Review According to the PRISMA Statement

**DOI:** 10.3390/jcm13041071

**Published:** 2024-02-14

**Authors:** Luigino Calzetta, Nicola Di Daniele, Alfredo Chetta, Marco Vitale, Shima Gholamalishahi, Mario Cazzola, Paola Rogliani

**Affiliations:** 1 Respiratory Disease and Lung Function Unit, Department of Medicine and Surgery, University of Parma, 43126 Parma, Italy; alfredoantonio.chetta@unipr.it; 2 Department of Systems Medicine, University of Rome “Tor Vergata”, 00133 Rome, Italy; didaniele@med.uniroma2.it; 3 Fondazione Leonardo per le Scienze Mediche Onlus, Policlinico Abano, 35031 Abano Terme, Italy; 4 Cardio-Thoracic and Vascular Department, University Hospital of Parma, 43126 Parma, Italy; 5 Faculty of Medicine, Vita-Salute University-San Raffaele, 20132 Milan, Italy; marco.vitale@unipr.it; 6 Italian Foundation for Research in Balneology (FoRST), 00198 Rome, Italy; 7 Unit of Respiratory Medicine, Department of Experimental Medicine, University of Rome “Tor Vergata”, 00133 Rome, Italy; shima.gholamalishahi@uniroma2.it (S.G.); mario.cazzola@uniroma2.it (M.C.); paola.rogliani@uniroma2.it (P.R.)

**Keywords:** asthma, COPD, systematic review, thermal water

## Abstract

Background: Asthma and chronic obstructive pulmonary disease (COPD) are global health challenges leading to substantial morbidity and mortality. While existing guidelines emphasize evidence-based treatments, the potential therapeutic role of thermal water (TW) inhalation remains under-investigated. Methods: This systematic review followed PRISMA-P guidelines and sought to evaluate the impact of TW in asthma and COPD. A thorough literature search, performed up to May 2023, encompassed in vitro, in vivo, randomized controlled trial (RCT), non-RCT, and observational studies. Results: The review included 12 studies reporting different findings. In vitro studies suggested TW could enhance antioxidant capacity and cell proliferation. In a murine model of non-atopic asthma, TW inhalation reduced airway hyperresponsiveness and inflammation. RCTs in COPD patients indicated mixed effects, including improved quality of life, reduced airway oxidant stress, and enhanced exercise tolerance. Asthma patients exposed to water aerosols exhibited improved lung function and reduced airway inflammation. Non-RCTs showed improved lung function and antioxidant activity after TW therapy. Additionally, observational studies reported enhanced lung function and reduced airway inflammation. Conclusion: The current evidence suggests potential benefits of TW therapy in asthma and COPD. However, limited high-quality RCTs and concerns regarding occupational TW exposure necessitate further investigation. While TW therapy offers a non-invasive treatment, its therapeutic potential still needs definitive demonstration. Future research should therefore prioritize well-designed RCTs to thoroughly establish the efficacy and safety of TW as a potential therapeutic intervention for asthma and COPD.

## 1. Introduction

Asthma and chronic obstructive pulmonary disease (COPD) are two chronic respiratory conditions with inflammatory characteristics that are the leading causes of morbidity and mortality worldwide, resulting in significant health care, economic, and social burdens, as well as reduced quality of life (QoL) [1]. Asthma and COPD are often associated with different mechanisms and symptoms of airway inflammation, airway obstruction, and chronic bronchitis [2]. In 2022, more than 3 million people died because of COPD, and it is predicted that the global burden of this disease will continue in the coming decades [3]. Asthma, the most prevalent chronic condition in childhood, burdens over a quarter of a billion individuals worldwide [2]. The Global Initiative for Asthma (GINA) and the Global Initiative for Chronic Obstructive Lung Disease (GOLD) are recommendations aimed to improve the understanding, management, and prevention of asthma and COPD, respectively [1,2]. Managing COPD and asthma requires a comprehensive approach to control symptoms, improve lung function, minimize risk factors, prevent exacerbations, and administer medications, oxygen therapy, and, potentially, surgical treatments. An important outcome is also to improve the overall QoL for individuals with these conditions [4].

For centuries, natural mineral waters have been utilized as a therapeutic treatment for different diseases [5]. Inhalation of thermal water (TW) is a traditional approach used for respiratory conditions such as COPD and chronic bronchitis. It entails inhaling aerosolized particles or steam from mineral-rich hot water [4]. However, it is worth noting that the last recommendations for managing these respiratory conditions do not include TW as potential management options. This is mainly because GINA and GOLD focus on evidence-based treatments supported by research and randomized clinical trials (RCTs), which, however, are scarce for TW [6]. The therapeutic use of TW offers several advantages, including non-aggressive treatment, minimal adverse events, and preventive properties, likely having beneficial effects including analgesic, antioxidant, antibacterial, and anti-inflammatory properties [5]. Thus, the effect of TW on asthma and COPD can be attributed to several key mechanisms.

To date, there has been no comprehensive systematic review addressing the influence of TW on asthma and COPD. Therefore, the aim of this study is to systematically review the current literature to provide evidence regarding the potential therapeutic impact of TW in asthma and COPD.

## 2. Methods

### 2.1. Review Question

The question of this systematic review was to assess the potential beneficial impact of TW in asthma and COPD, according to the evidence originating from research ranging from pre-clinical studies to RCTs.

### 2.2. Search Strategy and Study Eligibility

**Figure 1:**
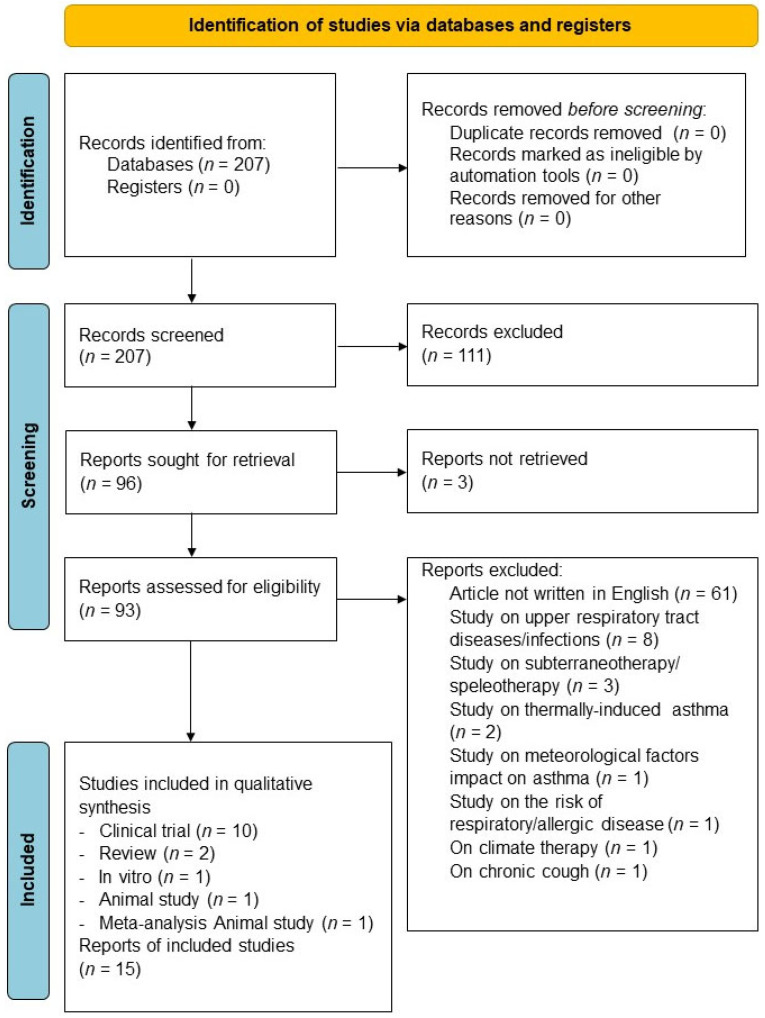
PRISMA flow diagram for the identification of studies included in the systematic review concerning the impact of TW on asthma and COPD. COPD: chronic obstructive pulmonary disease; PRISMA: Preferred Reporting Items for Systematic Reviews and Meta-Analyses; TW: thermal water.

The protocol of this synthesis of the current literature was submitted to PROSPERO (submission ID: 377191) and performed in agreement with the Preferred Reporting Items for Systematic Reviews and Meta-Analysis Protocol (PRISMA-P) [7], with the relative flow diagram shown in Figure 1. This study satisfied all the recommended items reported by the PRISMA-P checklist [7]. The PICO (Patient problem, Intervention, Comparison, and Outcome) framework was applied to develop the literature search strategy and question, as previously reported [8]. Namely, the “Patient problem” included asthma or COPD; the “Intervention” regarded the exposure to and/or treatment with TW; the “Comparison” was performed with respect to baseline and/or control; and the assessed “Outcome” was the impact on airway hyperresponsiveness (AHR) of methacholine (MCh), dyspnea, exercise capacity, health status, inflammation, oxidative stress, pulmonary function, QoL, symptoms control, and cell proliferation.

A comprehensive literature search was performed for in vitro studies, in vivo studies on research animals, clinical trials, meta-analyses, and reviews of literature evaluating the impact of TW in asthma or COPD. The search was performed in MEDLINE in order to identify relevant studies written in English and published up to 20 May 2023. The research string was as follows: ((thermal OR mineral) AND (water OR spring OR hydrotherapy OR balneotherapy)) AND (COPD OR asthma). Two reviewers (LC and SG) independently checked the relevant studies identified from the literature search. Citations of previous published relevant reviews and meta-analyses were examined to identify further pertinent studies, if any [5,9,10]. Literature search results were uploaded to Eppi-Reviewer 4 (EPPI-Centre Software. London, UK), a web-based software program for managing and analyzing data in literature reviews that facilitates collaboration among reviewers during the study selection process.

### 2.3. Data Extraction

Data from the included studies were extracted from published papers and/or supplementary files and checked for year of study and publication, type of study, type and number of cells, animals, cell donors and analyzed patients, type of exposure and/or treatment with TW, route of administration, age and gender of cell donors, experimental animals and analyzed patients, smoking habits, post-bronchodilator forced expiratory volume in the 1st s (second) (FEV_1_), investigated outcomes, and study quality assessment, via the Jadad Score and Cochrane Risk of Bias 2 (RoB 2) [11]. Data were extracted in agreement with Data Extraction for Complex Meta-analysis (DECiMAL) recommendations [12].

### 2.4. Endpoint

The endpoint of this systematic review was the effect of TW in asthma and COPD from pre-clinical and clinical evidence.

### 2.5. Strategy for Data Synthesis

Data from original papers were extracted and reported via qualitative synthesis.

### 2.6. Quality of Studies and Risk Bias

The summary of the risk of bias for each included randomized trial was analyzed via the Cochrane RoB 2 [11] and Jadad score [13]. The weighted assessment of the overall risk of bias was analyzed via the Cochrane RoB 2 [11] using the robvis visualization software [14,15].

The Jadad score, with a scale of 1–5 (score of 5 being the best quality), was used to assess the quality of the clinical trials concerning the likelihood of bias related to randomization, double blinding, withdrawals, and dropouts. The quality of the studies was assessed as follows: total score <3, low quality; total score =3, medium quality; total score >3, high quality. Two reviewers (LC and SG) independently assessed the quality of individual studies, and any difference in opinion about the quality score was resolved by consensus.

## 3. Results

### 3.1. Study Characteristics

Of the 207 potentially relevant records identified in the initial search, 12 studies were deemed eligible for a qualitative synthesis (Table 1). This systematic review included data obtained from five RCTs [4,16,17,18,19] evaluating the impact of TW on COPD [4,16,17,19] and pediatric asthma [18], two non-randomized, uncontrolled interventional studies [20,21] on asthma, two observational studies [22,23] on heavy smokers [22] and COPD patients [23], one case study [24] on workers in thermal-mineral springs exposed to sulfurous TW, one in vitro study [25] on human lung fibroblasts, and one in vivo study [26] on a murine model of non-atopic asthma. When reported in the studies, the temperature of the TW was defined as “warm” or “hot” and ranged from 37 to 80 °C. Data on viscosity and density of TW were not provided in the included studies.

### 3.2. Studies In Vitro

One in vitro study [25] investigated the impact of mineral waters (MWs) from Ledesma, Paracuellos, and Achena Spanish health spa resorts on human lung embryonic fibroblasts LC5. The three MWs were characterized by different chemical compositions: Ledesma MW was hyper-thermal, weakly mineralized, and rich in bicarbonate, chloride, sodium, and fluorides; Paracuellos MW was hypothermal, strongly mineralized, and described as chlorinated-sulphated-sodic; Archena MW was hyper-thermal, highly mineralized, and chlorinated-sodic [25]. Lung fibroblasts were cultured in medium diluted 10%, 20%, and 40% with MW treatments collected in spring and autumn and compared to control fibroblasts cultured with the same working dilutions in tap water. After 48 h of incubation, cell proliferation significantly (*p* < 0.05) increased in the presence of 10%, 20%, and 40% Achena MW collected in spring and autumn, 20–40% Paracuellos spring and autumn MW, and Ledesma MW dilutions of 20% from autumn and 40% from spring, compared to controls. When fibroblasts were treated with 40% Paracuellos and Achena MWs, cell proliferation exceeded the proliferation in controls by more than 50% (Paracuellos MW from spring: 168.66 ± 2.70%; Paracuellos MW from autumn: 185.54 ± 4.65%; Achena MW from spring 160.02 ± 3.46%; Achena MW from autumn: 185.26 ± 3.72%). Cell proliferation increased with rising MW concentration, except for Ledesma MW, which was weakly mineralized, and significantly (*p* < 0.05) increased in the presence of MW collected in autumn rather than spring, with the exception of 40% Ledesma MW [25].

An improved antioxidant activity was detected in MW-treated fibroblasts vs. controls. Extracellular reactive oxygen species (ROS) and reactive nitrogen species (RNS) levels were significantly (*p* < 0.05) greater in the presence of MW than in controls, except for Paracuellos spring MW and Achena autumn MW, suggesting an improved release of ROS-RNS to the extracellular medium, with a lower concentration detected in supernatants. There was no difference in ROS-RNS levels between spring and autumn MW-treated fibroblasts, apart from Ledesma MW (intracellular concentration: 1.46 ± 0.10 vs. 1.89 ± 0.18 mM; extracellular concentration: 0.72 ± 0.06 vs. 0.82 ± 0.03 mM). Reduced glutathione (GSH)/total GSH rate was significantly (*p* < 0.05) greater in fibroblasts cultured with Paracuellos spring and autumn MWs and with Achena autumn MW, compared to controls. The antioxidant capacity of only Paracuellos MW was found to be significantly (*p* < 0.05) lower in autumn than in spring (2.47 ± 0.14 vs. 2.29 ± 0.14 μM). No significant (*p* > 0.05) difference was detected in superoxide dismutase (SOD) 3 activity between MW-treated fibroblasts and controls [25]. Since cell proliferation was higher in the presence of autumnal MW, the authors decided to investigated cytokine secretion in these fibroblast cultures. Macrophage migration factor (MIF), Serpin E1, and interleukin (IL)-6 were released by both MW-treated and control fibroblasts, although cells cultured with Ledesma and Paracuellos MWs induced the highest level of secretion of Serpin E1 and IL-6 and released CCL1, CCL5, and ICAM-1. The cytokines IL-18 and CXCL-12 were released only by MW-treated fibroblasts, not by controls, and the release of CD40 and IL-13 was inhibited in the presence of Ledesma and Paracuellos MWs [25]. The main findings concerning the impact of MW in vitro are summarized in Table 2.

### 3.3. Studies in Experimental Animals

In a murine model of non-atopic asthma, Zajac et al. [26] investigated the mechanisms underlying the beneficial effects of inhalations of brine solution from the Wieliczka Salt Mine, a Polish health resort. Brine is a type of TW presenting a prevalence of sodium and chloride ions, as well as magnesium and calcium. In BALB/c mice skin-sensitized with 1-fluoro-2, 4-dinitrobenzene (DNFB) and challenged with cognate hapten, 12 inhalation sessions with brine solution significantly (*p* < 0.05) reduced the AHR to MCh 20–40 mg/mL compared to baseline. The level of AHR did not significantly (*p* > 0.05) change in response to increasing concentrations of MCh 5–40 mg/mL in brine-treated sensitized mice, but it was significantly (*p* < 0.05) lower compared to the untreated group. Brine solution inhalations significantly (*p* < 0.05) reduced the total inflammatory cell count and the number of neutrophils to a level comparable to that in sham-sensitized mice [26]. Concerning airway inflammation, the overexpression of IL-1α and IL-6 was significantly (*p* < 0.05) reduced in the lung tissue and bronchoalveolar lavage fluid (BALf) of brine-treated mice and the concentrations in lung tissue decreased to values observed in the sham-sensitized group. No modulatory effect was detected on IL-10 concentration in lung tissue and on IL-1β in the BALf of brine-treated sensitized mice. The overexpression of glutathione was significantly (*p* < 0.05) reduced in mice that inhaled brine solution but not in those administered with pure water or physiological saline [26]. Taken together, inhalations of brine solutions induced a reduction of AHR to MCh, an anti-inflammatory action at the cellular and cytokine levels, and a restored redox imbalance via the glutathione system [26]. The main findings concerning the impact of TW in laboratory animals are summarized in Table 3.

### 3.4. RCTs

Guarnieri et al. [4] performed a crossover RCT to evaluate whether salt-bromide-iodine TW inhalation treatment may induce biochemical modifications of the airway-lining fluid in COPD patients. A two-week course of TW therapy decreased (*p* = 0.05) the pH value of non-deaerated exhaled breath condensate (EBC) vs. baseline (median 7.45, IQR 6.93–7.66 vs. 6.99, IQR 6.57–7.19), suggesting that TW could cause an imbalance of volatile components in the airway-lining fluid. However, the treatment did not reduce the EBC concentration of the neutrophil chemoattractant leukotriene B4 (LTB4). TW id not significantly (*p* > 0.05) improve FEV_1_ and the dyspnea score, although the study was not powered to investigate clinical and functional outcomes [4].

Contoli et al. [17] evaluated the impact of sulfurous TW on airway oxidant stress and clinical outcomes in a double-blind RCT involving moderate to severe COPD patients. Twelve days of treatment with TW significantly (*p* < 0.001) inhibited the production of O_2_^−^ in the EBC, a condition that persisted for one month of follow-up. Sulfurous TW also significantly (*p* < 0.05) improved the COPD Assessment Test (CAT) score at one month follow-up. TW therapy did not affect either the total sputum inflammatory cell counts, or lung function assessed via FEV_1_ and residual volume [17]

A more recent RCT [16] compared the impact of two-week mud bath therapy with 12 sessions of leisure thermal activity in thermal pools on mild to severe COPD patients. Mud bath therapy included mud pack application followed by a bath in sulfurous TW, while leisure thermal activity comprised unsupervised activities in the thermal pools such as bathing, walking, and swimming. Upon challenge with inspiratory resistive breathing (IRB) at 40% of maximum inspiratory pressure, mud bath therapy significantly (*p* < 0.05) improved endurance time by 4.5 min compared to the −2.3 min decrease found post leisure thermal activity. Mud bath therapy did not significantly (*p* > 0.05) modulate the plasma level of IL-6 compared to leisure thermal activity. The change in IL-6 concentration after mud bath therapy was found to be predictive of higher O_2_ expenditure of the respiratory muscles’ endurance (VO_2Endur_) exclusively in patients allocated to mud bath therapy [16].

Gaisberger et al. [18] investigated the impact of ionized water aerosols on pediatric allergic asthma in a RCT. Children spent three weeks in an alpine summer asthma camp; half of them were exposed to water aerosols of an alpine waterfall for 1 h per day, while the other half spent the same time at a control site 2.3 km from the water aerosol group and at the same altitude. Exposure to water aerosol significantly (*p* < 0.05) improved the lung function parameters FEV_1_, FEV_1_/forced expiratory volume (FVC), forced expiratory flow (FEF) at 25% FVC (FEF_25_), FEF_50_, and maximum mid-expiratory flow over the middle half of the FVC (MMEF_25/75_). Peak expiratory flow (PEF) significantly (*p* < 0.01) improved only in the control group. The Asthma Control Test (ACT) symptom score significantly (*p* < 0.05) improved in both groups, but the change from baseline was significantly (*p* < 0.05) greater in the water aerosol group than in the control group. The fractioned nitric oxide (FeNO) level was significantly (*p* < 0.001) reduced both in children exposed to water aerosol and in controls (−44.4% and −53.6%, respectively). The number of IL-5-, IL-10-, and IL-13-producing peripheral blood mononuclear cells (PBMCs) derived from all children at the asthma camp significantly (*p* < 0.05) decreased in the water aerosol group. Gene expression of IL-13 in PBMCs was significantly (*p* < 0.05) reduced compared to controls, while there was no difference between the two groups in terms of IL-10 and interferon (IFN)-γ gene expression [18]. The level of eosinophil cationic protein (ECP) did not appear to be significantly modulated, while the number of regulatory T cells significantly (*p* < 0.05) increased in all children, with no significant (*p* > 0.05) difference between the water aerosol and control groups [18].

Pellegrini et al. [19] conducted a RCT on stable COPD patients to evaluate the impact of salt-bromine-iodine TW on airway inflammation, quality of life, dyspnea, and exercise capacity. Treatment with TW inhalations did not modulate lung function, 6 min walking test (6 MWT), or dyspnea score, compared to control. Saint George’s Respiratory Questionnaire (SGRQ) score showed a significant improvement upon TW treatment (from 28.0 ± 3.0 to 22.0 ± 3.0) vs. the control group (from 34.0 ± 3.0 to 38.0 ± 4.0). In induced sputum, total inflammatory cell counts significantly (*p* < 0.05) increased in both the TW group (from median 2881 cells/mg, IQR 1655–4786 to 2964 cells/mg, IQR 1944–9218) and the control group (from median 1776 cells/mg, IQR 1104–2591 to 2700 cells/mg, IQR 2161–3958). Inhalation of TW produced a small and significant (*p* < 0.01) reduction in the percentage of sputum neutrophils (from median 64.3, IQR 50–78 to 61.8, IQR 47–71) and an increase in macrophages (from median 34.1, IQR 22–49 to 37.6, IQR 29–52) [19]. No other significant difference was detected in the number of differential cells [19]. The main findings concerning the impact of TW in RCTs are summarized in Table 4.

### 3.5. Non-RCTs

A pilot clinical study [20] evaluated the clinical effects of radon-containing TW inhalation therapy performed once a week for one month on asthmatic patients in relation to antioxidant enzymes and lipid peroxide. TW significantly (*p* < 0.05) improved FEV_1_% predicted, and numerically increased FVC% and FEV_1_/FVC vs. baseline. The activities of blood catalase and SOD were significantly (*p* < 0.05) increased vs. control (a blood sample collected before therapy), while the level of lipid peroxide significantly (*p* < 0.05) decreased [20].

Tanizaki et al. [21] investigated the clinical effects of spa therapy, including swimming training in a TW spring pool, inhalation of iodine salt solution, and mud therapy in steroid-dependent intractable asthma. TW therapy was effective in 69.2% of patients, meaning that the treatment induced a slight, moderate, or marked improvement in asthma attacks, dyspnea, and the glucocorticoid dose. The efficacy was higher in patients aged 41–50 years (87.5%) and 51–60 years (84.2%), compared to the other age groups. The efficacy was greater in patients having bronchoconstriction with an amount of expectoration 50–99 mL/day (83.4%), in those showing bronchoconstriction and hypersecretion <100 mL/day (77.8%), and in those with bronchiolar obstruction (80.0%), than in patients with bronchoconstriction and an amount of expectoration of 0–49 mL/day. Efficacy appeared to be associated with airway inflammation, as the BALf neutrophil and eosinophil counts were higher in patients with a marked or moderate improvement compared to those with slight or no improvement. TW treatment induced a significant (*p* < 0.05) improvement in both MMEF and %V_25_ by >20% in all patients except for those displaying simple bronchoconstriction and expectoration of 0–49 mL/day. AHR to MCh was significantly (*p* < 0.05) inhibited after TW therapy in all patients [21]. The main findings concerning the impact of TW in non-RCTs are summarized in Table 5.

### 3.6. Observational Studies

Carubbi et al. [22] investigated the impact of ten-day-inhalation treatment with sulfurous TW on pulmonary function and inflammation in current and former heavy smokers. TW inhalation therapy did not modulate spirometric parameters, but a significant (*p* < 0.05) increase in citrulline and a decrease in ornithine levels was observed in EBC, thus shifting the metabolism of arginine towards the reduction in nitric oxide production and displaying an anti-inflammatory phenotype [22].

Takata et al. [23] evaluated the beneficial effect of four-week spa therapy in hospitalized COPD patients undergoing pulmonary rehabilitation. An improvement, although not significant, was observed in vital capacity (VC), FEV_1_, and 6 MWT. Maximum Borg scale values after TW therapy were significantly (*p* < 0.05) reduced from 2.0 ± 1.3 to 1.6 ± 1.3, thus demonstrating a better disease control.

A case study [24] of Contursi Terme (Salerno, Italy) investigated the extent of exposure to sulfurous compounds of workers in thermal-mineral springs. Although TW is known to be beneficial for human health, the atmosphere of such workplaces is characterized by the presence of potentially toxic compounds including hydrogen sulfide (H_2_S) and SO_2_. After four months of air and TW monitoring, it was found that the air concentration of SO_2_ ranged between 0.11 ± 0.02 and 0.91 ± 0.02 mg/m^3^, according to a seasonal pattern (higher values in winter and lower in spring). Indoor H_2_S level did not significantly vary over time, but outdoor levels (between 0.40 ± 0.03 and 1.90 ± 0.03 mg/m^3^) were always higher than indoor values (between 0.11 ± 0.03 and 0.56 ± 0.03 mg/m^3^) [24]. The main findings concerning the impact of TW in observational studies are summarized in Table 6.

## 4. Quality of Evidence and Risk of Bias

Two of the five RCTs (40.00%) included in this systematic review were ranked as being of medium to high quality (Jadad score ≥ 3), whereas the other 3 (60.00%) were characterized by a low-quality level (Jadad score < 3).

**Figure 2:**
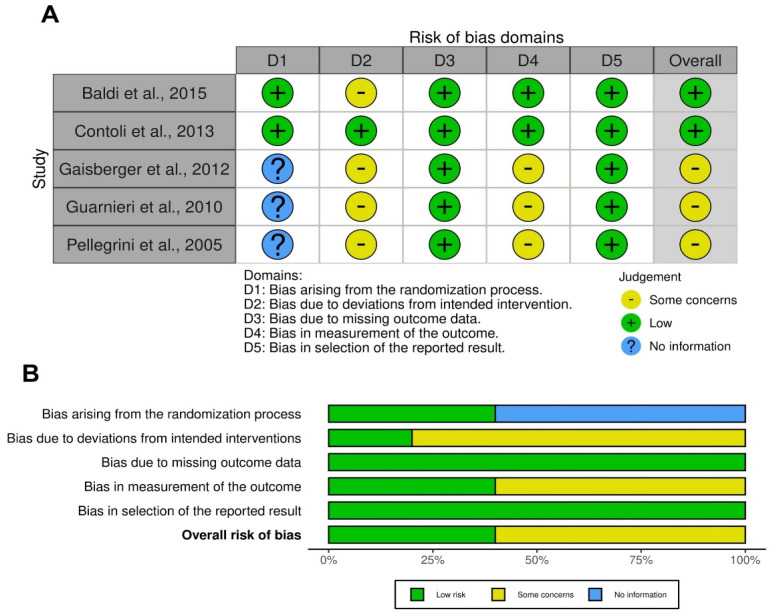
Traffic light plot for assessment of the risk of bias of each included RCT (**A**) and weighted plot for the assessment of the overall risk of bias (**B**) via the Cochrane RoB 2 tool (*n* = 5 studies, from 5 records). Traffic light plot reports five risk-of-bias domains: D1, bias arising from the randomization process; D2, bias due to deviations from intended intervention; D3, bias due to missing outcome data; D4, bias in measurement of the outcome; D5, bias in selection of the reported result. Yellow circle indicates some concerns regarding the risk of bias, green circle represents low risk of bias, and blue circle indicates insufficient information about the risk of bias. RCT: randomized controlled trial; RoB: risk of bias.

The traffic light plot for the assessment of the risk of bias of each of the included clinical studies is reported in Figure 2A, and the weighted plot for the assessment of the overall risk of bias by domain is shown in 2B. All the RCTs (five, 100.0%) had a low risk of bias for missing outcome data and selection of the reported result; two studies (40.0%) showed a low risk of bias for the randomization process and measurement of the outcome; and one study (20.0%) had a low risk of bias in the domain of deviations from intended interventions. Four RCTs (80.0%) provided some concerns regarding the risk of bias due to deviations from intended interventions, and three RCTs (60.0%) regarding the measurement of the outcome. Three studies out of five (60.0%) had no information about the bias arising from the randomization process.

## 5. Discussion

The results of this systematic review suggest that TW may have some beneficial effect in asthma and COPD.

The outcomes of in vitro evidence reported a positive effect on human lung fibroblasts, including increased levels of GSH and antioxidant capacity (ROS-RNS) [25]. In vivo findings from a murine model of non-atopic asthma indicated that brine inhalations had a positive effect on AHR and inflammatory cells [26].

Concerning RCTs carried out in COPD patients, TW treatment by salt-bromide-iodine not only decreased pH and provided anti-inflammatory effects on the airways, but also improved the overall health-related QoL [4,19]. Additionally, sulfurous TW exhibited a positive impact on reducing O_2_^−^ production, with no effect on total sputum inflammatory cell counts and FEV_1_ [17]. The mud bath therapy demonstrated its positive influence on enhancing tolerance and endurance time, oxygen expenditure, and effectively addressing moderate IRB challenges [16]. With respect to RCTs on asthmatic patients, water aerosol has been shown to improve FEV_1_, asthma control, and airway inflammation [18].

Some non-RCTs have reported that radon, thermal, and spa therapies can have beneficial effects on asthma and lung function [20,21]. These therapies have been observed to increase the levels of SOD, an antioxidant enzyme that protects against oxidative stress. In addition, these treatments have been associated with a decrease in lipid peroxide levels [20,21]. Based on the results of two observational studies conducted on heavy smokers, it has been observed that sulfurous TW has a beneficial impact on some inflammatory factors in the airways of smokers. However, there is still limited evidence regarding the efficacy of this treatment [20,22]. Furthermore, improvement in ventilatory parameters has been observed in patients with COPD after undergoing spa therapy [21].

Two previous narrative reviews [5,10] have reported the potential benefits of sulfurous TW therapy in the inhalational treatment of asthma and COPD. These articles suggest that this treatment has the potential to improve the QoL in patients affected by these diseases [5,10]. Despite such positive evidence, TW may have some detrimental impacts. According to the results of in vitro evidence, an unfavorable effect of TW may be observed due to the increased release of cytokines and cell proliferation [25]. Moreover, inhalation of TW was found to enhance the number of macrophages in patients with COPD [19] and the number of neutrophils and eosinophils in asthmatic subjects [18].

Although the water of thermal structures provides beneficial effects on human health, the atmosphere of these environments may be characterized by the presence of sulfurous compounds such as H_2_S and SO_2_ [24]. H_2_S and SO_2_ are important air pollutants that are dangerous for worker safety [24]. In the case study conducted by Pironti et al. [24], it was found that concentrations of sulfurous compounds, specifically H_2_S and SO_2_, exceeded the permissible limits in the working environment for certain workers [24]. The quality of the air in workplaces has generally improved over the past few years but preserving workers’ health remains a concern.

According to the latest global epidemiological studies, in 2019, approximately 262 million people worldwide were affected by asthma, and an additional 212 million by COPD [27,28]. Concerning asthma, its prevalence in the European Union (EU) is estimated to be 8.2% in adults and 9.4% in children [29]. More specifically in Italy, where most of the studies on TW have been carried out, the overall prevalence of asthma is 6.10% [30]. This means that around one worker in every twelve in a TW structure are asthmatic patients at risk of AHR and asthma exacerbation due to the exposure to H_2_S and SO_2_ [31,32]. Therefore, chronic exposure to H_2_S and SO_2_ may be a relevant epidemiological concern for subjects working in structures for TW therapy [31]. Since it is not possible to rule out the potential detrimental impact of exposure to chronic sulfurous compounds, further assessment of occupational risks and implementation of measures to ensure the safety of workers in that specific workplace are needed.

Considering the limitations of the available evidence, it is important to note that the current body of research is characterized by a few RCTs, with the majority of these studies exhibiting low methodological quality. Only the studies of Baldi [16] and Contoli [17] achieved a medium-to-high quality rating. Certainly, this represents the main intrinsic limitation of this systematic review. Furthermore, evidence from a previous meta-analysis is currently available [9], but the effect estimate resulted in a quantitative synthesis characterized by low-quality evidence and, thus, conclusions should be interpreted with caution.

In any case, TW therapy has been suggested as a potentially beneficial add-on treatment to pharmacological interventions for reducing the frequency of exacerbations and infections by stimulating the natural local defense mechanisms in the airways [10]. However, these beneficial effects lack evidence-based proof. Additionally, most clinical trials investigating the use of TW in asthma and COPD do not provide details about concurrent pharmacological treatments. In some cases, it is even challenging to discern whether TW inhalations were administered as add-on therapies [10].

Overall, the current evidence on the impact of TW on asthma and COPD is weak. Well-designed RCTs are strongly needed to thoroughly assess whether TW may indeed have beneficial effects in these chronic obstructive respiratory disorders.

## Figures and Tables

**Table 1:** Characteristics of the studies included in the systematic review.

Study, Year and Reference	Type of Study (Characteristics)	Treatment Duration	Type of Cells, Animals, Donors, or Analyzed Patients	Number of Cell Donors, Animals, or Analyzed Patients	Type of Exposure and/or Treatment with TW	Route of Administration and Temperature	Age (Years)	Male (%)	Current Smokers (%)	Post Bronchodilator FEV_1_ (% Predicted)	Comparator	Evaluated Outcomes	Jadad Score
Pironti et al., 2022 [24]	Clinical trial (case study)	4 months	Workers in thermal-mineral springs	NA	Bicarbonate sulfurous TW from Contursi Terme in Salerno, Italy	NA,” hot”	NA	NA	NA	NA	NA	Physicochemical analysis of TW and monitoring of air concentrations of H_2_S and SO_2_ in the thermal springs	NA
Zajac et al., 2020 [26]	In vivo study (murine model of non-atopic asthma)	NA	BALB/c mice intratracheally sensitized with 0.5% DNFB and challenged with cognate hapten-50 μL of 0.6% DNS	50	Brine solution containing sodium chloride TW from the “Wieliczka” Salt Mine	Inhalation, NA	7–8 wks	100.0	NA	NA	Sensitized mice inhaled with pure water or 0.9% NaCl or untreated	AHR, inflammation, and oxidative stress	NA
Carubbi et al., 2019 [22]	Clinical trial (observational, cohort study)	10 days	Heavy current and former smokers with ≥20 pack-years of smoking history	419	Sulfurous TW	Inhalation, NA	63.2	58.9	49.2	87.4	Baseline	Pulmonary function and EBC metabolic analysis	NA
Melgar-Sánchez et al., 2019 [25]	In vitro study	NA	Human lung embryonic fibroblast LC5 cells	NA	Sulfurous MW from Spanish health resorts of Baños de Ledesma, Paracuellos de Jiloca, and Archena	Incubation, NA	NA	NA	NA	NA	Untreated cells	Cell proliferation, oxidative stress, and inflammation	NA
Baldi et al., 2015, NCT01253941 [16]	Clinical trial (single-center, randomized, unblinded, parallel-group study)	2 wks (12 sessions)	Mild to severe COPD patients (FEV_1_/FVC ≤ 70%, change in post-bronchodilator FEV_1_ < 12% and 200 mL defined the presence of chronic airflow obstruction)	42	Sulfurous TW from Colli Euganei mineral-water field	Exposure to TW by unsupervised leisure activity in thermal pools, 38–42 °C	64.6	78.6	40.5	68.3	Mud bath therapy	Pulmonary function, endurance time and O_2_ expenditure, and plasma IL-6 concentration level following an IRB challenge	3
Contoli et al., 2013, NCT01664767 [17]	Clinical trial (single-center, randomized, controlled, double-blind, parallel-group study)	12 days	COPD patients (FEV_1_/FVC < 70%, post-bronchodilator FEV_1_ > 30% and < 80% of predicted; at GOLD stages 2 and 3)	40	Sulfurous TW from Terme di Riolo in Ravenna, Italy	Inhalation, “warm”	69.9	72.5	5.0	58.1	Inhalation of isotonic saline solution	Pulmonary function, health status, oxidative stress, and airway inflammation	5
Gaisberger et al., 2012, ISRCTN04002573 [18]	Clinical trial (single-center, randomized controlled, unblinded, parallel-group study)	3 wks, 1 h per day	Children affected by asthma	54	Exposure to waterfall environment by inhaling of ions and aerosols generated by splashing of water termed “ionosols” combined with high-altitude climate therapy	TW aerosol by exposure to waterfall, NA	11.0	69.2	0.0	81.8	High-altitude climate therapy	Pulmonary function, symptoms control, airway inflammation (including F_e_NO)	2
Guarnieri et al., 2010 [4]	Clinical trial (single-center, randomized, controlled, single-blind, crossover study	2 wks	COPD patients (FEV_1_/FVC < 70%, post-bronchodilator reversibility < 12% and 200 mL of initial FEV_1_; at GOLD stages 1–3)	13	Salt-bromide-iodine TW	Inhalation, 80 °C	69.0	76.9	38.5	66.6	Inhalation of normal saline and baseline	Airway-lining fluid by analysis of EBC pH and LTB_4_ level, pulmonary function, and dyspnea	2
Takata et al., 2008 [23]	Clinical trial (observational study)	4 wks	Hospitalized COPD patients (GOLD stages 1–3)	25	Spa therapy including swimming training and exercise in a hot spring pool, inhalation of iodine salt solution and fango therapy	Exposure to environment with TW and inhalation, “hot”	73.3	88.0	0.0	NA	Baseline	Pulmonary function, 6 MWT, and O_2_ saturation	NA
Pellegrini et al., 2005 [19]	Clinical trial (single-center, randomized, controlled, single-blind, parallel-group study)	2 wks	Stable COPD (FEV_1_/FVC < 70%, post-bronchodilator reversibility < 15% or 200 mL of initial FEV_1_; patients with smoking history of ≥10 pack/year and chronic bronchitis)	39	Salt-bromide-iodine TW	Inhalation, 37 °C	64.0	89.7	100.0	72.0	Inhalation of normal saline and baseline	Inflammation, SGRQ, dyspnea, and 6 MWT	2
Mistunobu et al., 2003 [20]	Clinical trial (pilot interventional study, not randomized, not controlled)	4 wks	Atopic asthma	9	Radon and TW therapy	Inhalation, 48 °C	59.0	55.6	0.0	NA	Baseline	Pulmonary function and oxidative stress	NA
Tanizaki et al., 1993 [21]	Clinical trial (interventional study, not randomized, not controlled)	1–3 months	Steroid-dependent intractable asthma	52	Exposure to TW by spa therapy including swimming training in a hot spring pool, iodine salt solution inhalation twice daily, and fango therapy	Exposure to environment with TW and inhalation, 70–80 °C	57.7	44.2	0.0	NA	Baseline	AHR, pulmonary function and inflammation	NA

AHR: airway hyperresponsiveness; COPD: chronic obstructive pulmonary disease; DNS: dinitrobenzene sulfonic acid; EBC: exhaled breath condensate; F_e_NO: fractioned nitric oxide; FEV_1_: forced expiratory volume in the 1st second; FVC: forced vital capacity; GOLD: Global Initiative for Chronic Obstructive Lung Disease; IL: interleukin; IRB: inspiratory resistive breathing; LTB_4_: leukotriene B4; MW: mineral water; NA: not available; O_2_: oxygen; RCT: randomized controlled trial; SGRQ: Saint George’s Respiratory Questionnaire; TW: thermal water; wks: weeks; 6 MWT: 6 min walking test.

**Table 2:** Impact of MW in vitro.

Type of MW	Outcomes			
	Cell Proliferation (Fibroblasts)	Oxidant Activity (ROS-RNS)	GSH	Cytokines Profiling (MIF, IL-6, CL-1, CCL-5, ICAM-1)
**Ledesma**	↑ [25]	≈ [25]	NA	↑ [25]
**Paracuellos**	↑ [25]	↑ in autumn ↓ in spring [25]	↑ in autumn and spring [25]	↑ [25]
**Archena**	↑ [25]	↑ in spring ↓ in autumn [25]	↑ in autumn [25]	↑ [25]

↑: increase; ↓: decrease; ≈: approximately equal; GSH: glutathione; MW: mineral water, NA: not available; ROS: reactive oxygen species; RNS: reactive nitrogen species.

**Table 3:** Impact of TW in experimental animals.

Type of TW	Outcomes							
	AHR to MCh	Inflammatory Cells	Neutrophil	IL-1α	IL-6	IL-10	IL-1β	GSH
**Wieliczka Salt Mine** **(inhalation of brine solution)**	↓ [26]	↓ [26]	↓ [26]	↓ [26]	↓ [26]	≈ [26]	≈ [26]	↓ [26]
**Polish health resort** **(inhalation of brine solution)**	↓ [26]	↓ [26]	↓ [26]	↓ [26]	↓ [26]	≈ [26]	≈ [26]	↓ [26]

↓: decrease; ≈: approximately equal; AHR: airway hyperresponsiveness; MCh: methacholine; TW: thermal water.

**Table 4:** Impact of TW in RCTs.

Type of Mineral Water	Outcomes																		
	LTB 4 in EBC	pH in EBC	FEV_1_	O_2_^−^ Production	CAT	ACT	Dyspnea	SGRQ	ECP	T Cell	ET	Sputum	Sputum Neutrophils	Macrophage	IRB	IL-6	VO_2Endur_	F_e_NO	IL-5-, IL-10, IL-13
**Salt-bromide-iodine** **(inhalation)**	≈ [4]	↓ [4]	≈ [4,19]	NA	NA	NA	≈ [4,19]	↓ [19]	NA	NA	NA	↓ [19]	↓ [19]	↑ [19]	NA	NA	NA	NA	NA
**Sulfurous** **(inhalation)**	NA	NA	≈ [17]	↓ [17]	↓ [17]	NA	NA	NA	NA	NA	NA	≈ [17]	NA	NA	NA	NA	NA	NA	NA
**Mud bath water** **natural clay** **sulfur-rich mineral**	NA	NA	NA	NA	NA	NA	NA	NA	NA	NA	↑ [16]	NA	NA	NA	↑ [16]	≈ [16]	↑ [16]	NA	NA
**Alpine waterfall**	NA	NA	↑ [18]	NA	NA	↑ [18]	NA	NA	≈ [18]	↑ [18]	NA	NA	NA	NA	NA	NA	NA	↓ [18]	↓ [18]

↑: increase; ↓: decrease; ≈: approximately equal; CAT: COPD Assessment Test; ACT: Asthma Control Test; EBC: exhaled breath condensate; ECP: eosinophil cationic protein; ET: endurance time; FEV_1_: forced expiratory volume in the 1st second; IRB: inspiratory resistive breathing; LTB_4_: leukotriene B_4_; SGRQ: Saint George’s Respiratory Questionnaire; VO_2Endur_: O_2_ expenditure of the respiratory muscle’s endurance; F_e_NO: fractioned nitric oxide; TW: thermal water.

**Table 5:** Impact of TW in non-RCTs.

Type of TW	Outcomes				
	FEV_1_/FVC	Blood Catalase and SOD	Lipid Peroxide	Neutrophil—Eosinophil	AHR
**Radon and TW** **(inhalation)**	↑ [20]	↑ [20]	↓ [20]	NA	NA
**Iodine salt solution (inhalation)**	NA	NA	NA	↑ [21]	↓ [21]

↑: increase; ↓: decrease; AHR: airway hyperresponsiveness; FEV_1_: forced expiratory volume in the 1st second; FVC: forced vital capacity; SOD: superoxide dismutase; TW: thermal water.

**Table 6:** Impact of TW in observational studies.

Type of TW	Outcomes						
	Citrulline	Ornithine	NO	FEV_1_	Maximum Borg Scale Values	SO_2_	H_2_S
**Sulfurous** **(inhalation)**	↑ [22]	↓ [22]	↓ [22]	NA	NA	↑ [24]	≈ indoor; ↑ outdoor [24]
**Spa therapy**	NA	NA	NA	≈ [23]	↓ [23]	NA	NA

↑: increase; ↓: decrease; ≈: approximately equal; H_2_S: hydrogen sulfide; NO: nitric oxide; SO_2_: sulfur dioxide; TW: thermal water.

## Data Availability

All data included in this systematic review are available in the primary publications.
